# Spatial–Temporal Heterogeneity and Driving Mechanisms of the Relationship Between Vegetation Carbon Sequestration and Biogenic Volatile Organic Compounds (BVOC) Emissions in China

**DOI:** 10.3390/plants15040564

**Published:** 2026-02-11

**Authors:** Yibing Li, Xiaoxiu Lun, Panfei Fang, Shaodong Huang, Yuying Liang, Yujie Li, Pengfei Zheng, Jia Wang, Longhuan Wang

**Affiliations:** 1 Beijing Key Laboratory of Precision Forestry, Beijing Forestry University, Beijing 100083, China; liyibing@bjfu.edu.cn (Y.L.); fangpanfei@126.com (P.F.); 4321hsd@gmail.com (S.H.); liangyy082844@163.com (Y.L.);; 2 Ministry of Education of Engineering Research Center for Forest and Grassland Carbon Sequestration, Beijing Forestry University, Beijing 100083, China; 3 College of Environmental Science and Engineering, Beijing Forestry University, Beijing 100083, China; lunxiaoxiu@bjfu.edu.cn; 4 Research Institute of Forestry Policy and Information, Chinese Academy of Forestry, Beijing 100091, China; zhengpengfei@swfu.edu.cn

**Keywords:** biogenic volatile organic compounds, gross primary productivity, spatial-temporal heterogeneity, driving mechanism, hydroclimatic variables

## Abstract

Vegetation plays a dual role in the Earth’s climate system: it removes atmospheric CO_2_ through photosynthesis while emitting biogenic volatile organic compounds (BVOCs), which can weaken the net carbon sink and contribute to air pollution. To assess the long-term interplay between carbon uptake and BVOC emissions, and to clarify how vegetation characteristics and climate regulate this relationship, we developed a Biogenic Carbon Efficiency Index (BCEI). The BCEI integrates BVOC emissions with gross primary productivity (GPP) to quantify their spatial ratio, thereby capturing the concurrent “source” and “sink” attributes of vegetation. We characterize the spatiotemporal heterogeneity of the BCEI across China and identify its dominant environmental drivers. The BCEI decreases from southeast to northwest, and during 2001–2020 exhibited a declining trend over 78% of the country, with increases mainly in Southwest China and on the Shandong and Liaodong Peninsulas. Driver analyses indicate that variables linked to hydrothermal conditions, including temperature, precipitation, evapotranspiration, and soil moisture, primarily control BCEI variability. Across most regions, the BCEI is negatively correlated with soil moisture and precipitation, positively correlated with evapotranspiration, and shows regionally varying associations with temperature. These findings deepen understanding of vegetation’s dual role as a source and sink and its driving mechanisms, providing a theoretical basis for optimizing regional vegetation management strategies.

## 1. Introduction

Vegetation plays a pivotal role in the global carbon cycle. As a major component of terrestrial carbon uptake and storage, it assimilates atmospheric CO_2_ through photosynthesis and thus mitigates the buildup of greenhouse gases [1]. However, vegetation simultaneously releases biogenic volatile organic compounds (BVOCs), a diverse group of volatile organic compounds produced and emitted by vegetation (e.g., isoprene and monoterpenes). These emissions contribute to the formation of tropospheric ozone and secondary organic aerosol (SOA), thereby affecting air quality and human health at regional-to-global scales [2,3]. Recent studies suggest that BVOC emissions from forests may offset up to one-third of their carbon sink benefits [4]. Focusing solely on vegetation’s carbon sink benefits while overlooking the adverse impacts of BVOC emissions can lead to a biased appraisal of vegetation’s contribution to climate regulation. BVOCs can promote tropospheric ozone formation and secondary organic aerosol production, which deteriorate air quality and affect climate forcing [5]. Accordingly, establishing the quantitative relationship between carbon uptake and BVOC emissions, together with their spatiotemporal variability, is prerequisite to robust impact assessment and underpins science-based greening and ecological restoration strategies.

Over the past three decades, China has played a pivotal role in global greening through large-scale afforestation and ecological restoration programs. Since the 1980s, the national leaf area index (LAI) has increased by roughly 10%, and forest area has expanded to 179.89 million hectares [6]. According to Cai et al. [7], China accounted for approximately 25% of the global gain in greening from 2000 to 2020, positioning it among the leading drivers of this trend. In parallel, China’s vegetation-mediated carbon sink intensified: plantation carbon stocks increased from 675.6 ± 12.5 Tg C (1990) to 1873.1 ± 16.2 Tg C (2020), equivalent to an average rise of about 40 Tg C per year [8]. Moreover, the greening trend across China’s vegetation has been pronounced, with an expansion rate nearly three times the global average [9]. At present, vegetation accounts for roughly 60% of the total terrestrial carbon sink in China [10,11]. With the continued implementation of greening policies, this proportion has risen to 74% [12,13].

However, the continued expansion of vegetated area and forest growing stock volume has raised concerns about a potential increase in China’s BVOC emissions. This concern is substantiated by multiple studies. As early as Li and Xie [14], national BVOC emissions were reported to have increased at an annual rate of 1.27% during 1981–2003. The upward trajectory persisted into the twenty-first century: Wang et al. [15] estimated a further 11.7% increase in total national emissions between 2001 and 2016. Most recently, Gai et al. [16] focused on key regions and found that BVOC emissions in several priority afforestation zones in China are still rising. Such sustained growth in emissions has been implicated as an important driver of the formation of ground-level ozone and PM2.5 (via secondary processes), posing an increasingly serious challenge to air quality and public health [17].

Despite substantial advances in quantifying the magnitude [18,19], trends [20] and drivers [21,22] of vegetation carbon sinks and BVOC emissions, evidence on their coupling in China remains scarce. In particular, the spatiotemporal relationship between BVOC emissions and carbon storage, including its variation with vegetation physiological status and environmental conditions, remains unresolved. Early work argued that BVOC emissions represent a loss of carbon previously fixed by the terrestrial biosphere and should therefore be accounted for in carbon budgeting. Kesselmeier et al. [23] argued that VOC (including BVOC) emissions are a major biogenic carbon loss pathway and called for more representative measurements linking emission fluxes to carbon storage. Building on this premise, Guo et al. [24] compared the contribution of carbon emitted as BVOCs to net primary productivity (BVOC/NPP) across subtropical urban–rural systems and found that this contribution was systematically higher in built-up areas than in rural forests, excluding bamboo forests. This pattern is plausibly linked to stronger urban environmental stresses (e.g., heat and pollution), which can enhance stress-induced BVOC emissions [25]. Across China’s climate zones, Bai et al. [26] identified a clear latitudinal pattern in BVOC/NEE, with maxima in the tropics and minima in Arctic and sub-Arctic regions. Mechanistically, BVOC emissions co-vary with photosynthesis: increases in GPP are often accompanied by elevated BVOC emissions [27].

GPP is used to represent vegetation carbon uptake because it reflects photosynthetic carbon assimilation at large spatial scales and avoids uncertainties associated with carbon allocation and respiration inherent in metrics such as NPP or NEP [28]. As many BVOCs are synthesized from recently assimilated carbon, GPP provides a consistent basis for linking carbon uptake with BVOC emissions. To account for differences in ozone-forming efficiency among BVOC species, ozone formation potential (OFP) is calculated using maximum incremental reactivity (MIR) rather than emission magnitude alone [29], allowing ozone-related air quality impacts to be evaluated relative to vegetation carbon uptake.

Two critical gaps remain. (i) Existing studies predominantly focus on site-level or city-specific systems and lack comprehensive national coverage; and (ii) most analyses focus on static associations between carbon sinks and BVOC emissions rather than their multi-scale co-evolution and mechanisms. In addition to rapid greening, China’s climate has shifted substantially over the last three decades, characterized by sustained warming and reorganized precipitation patterns [30,31]. Together, greening and climate change modulate BVOC emissions, which in turn affect the carbon cycle via complex atmospheric chemical pathways. However, systematic evaluations of spatial heterogeneity and dominant drivers are scarce, limiting robust estimates of vegetation’s net climatic benefits and impeding insight into coupled land–atmosphere processes. A national-scale, spatiotemporal assessment linking BVOC emissions and carbon sink dynamics is thus warranted.

**Figure 1:**
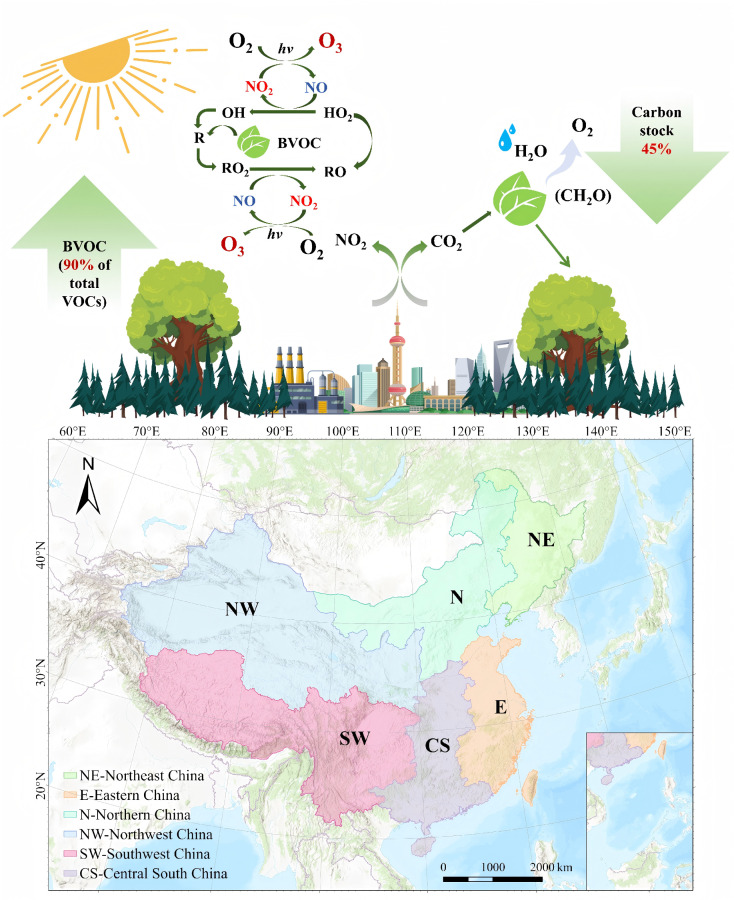
Conceptual framework of vegetation carbon uptake, BVOC emissions, and ozone formation, together with the regional division of the study area in China.

To systematically evaluate the spatiotemporal coupling between vegetation carbon sinks and BVOC emissions, we develop an integrated metric, the Biogenic Carbon Efficiency Index (BCEI). The BCEI explicitly quantifies the ozone formation potential per unit of carbon fixed by vegetation, providing a unified indicator of the trade-off between ecosystem carbon sequestration and air quality impacts, thereby quantifying the trade-off between carbon uptake and atmospheric pollution. Specifically, we (i) analyze the evolution and spatial pattern of the BCEI across China during 2001–2020 to identify long-term trends and potential hotspots; (ii) partition the domain into six ecogeographical regions (Northeast, North China, Northwest, East China, Central–South, and Southwest) and assess regional BCEI dynamics to reveal spatial heterogeneity in carbon–pollution coupling; and (iii) apply the Geographical Detector framework to integrate climatic variables, vegetation structure, and land use factors, identifying dominant drivers of the BCEI and comparing their explanatory power across regions to elucidate underlying mechanisms. By coupling a quantitative index with a multi-scale analytical design, this study characterizes the dynamic balance between vegetation carbon sequestration and BVOC emissions, providing an evidence base for region-differentiated ecosystem management and for optimizing greening policies under China’s “dual-carbon” agenda. To provide an intuitive overview of the conceptual framework and the spatial scope of this study, Figure 1 illustrates the coupling between vegetation carbon uptake and BVOC-driven ozone formation, together with the regional division of the study area across China.

## 2. Results

### 2.1. Temporal Change Trends of BCEI in Different Scales

**Figure 2:**
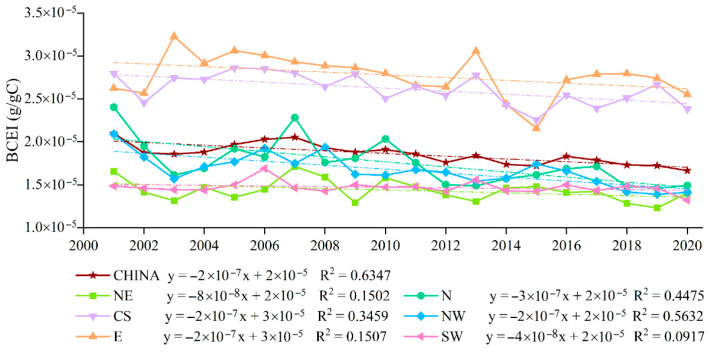
Trends in annual average BCEI for the different regions, 2001–2020. BCEI denotes the Biogenic Carbon Efficiency Index. Abbreviations: NE, Northeast; N, North; NW, Northwest; E, East; CS, Central–South; SW, Southwest. Dashed lines indicate the corresponding linear regression trends for each region.

Between 2001 and 2020, the BCEI exhibited an overall downward trend across China, with values ranging from 0.0000166 g/g C to 0.000021 g/g C. Figure 2 illustrates the temporal variations in the BCEI in the six major regions of China during this period. The results indicate that all regions showed downward trends to varying degrees, exhibiting spatial differences. In terms of regional averages, the mean BCEI values in the Eastern (E) and Central–South (CS) regions were higher than in other regions over the 20-year period. The Eastern China (E) region had the highest annual mean BCEI, with a peak value of 0.0000323 g/g C, while the Northeast (NE) and Southwest (SW) regions had the lowest mean values, with a maximum of only 0.000017 g/g C. Regarding the trends, all six regions showed a consistent downward trajectory for the BCEI, although the rates of decline varied significantly.

### 2.2. Spatial Distribution of BCEI

**Figure 3:**
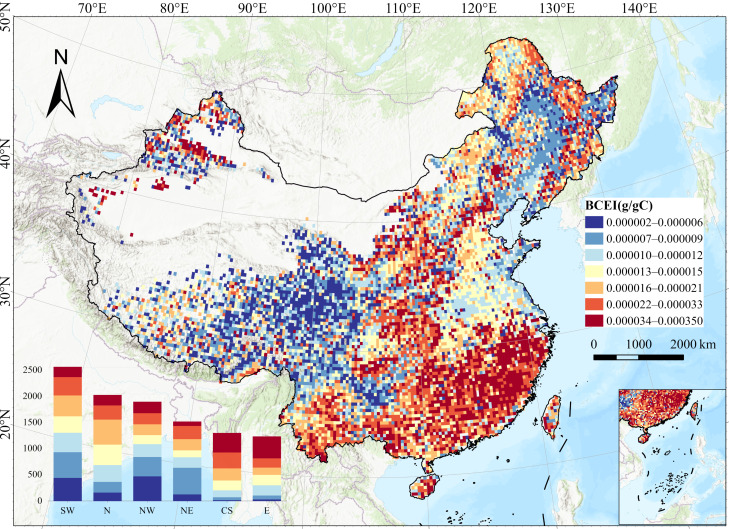
Spatial distribution of the average annual BCEI in China from 2001 to 2020. BCEI denotes the Biogenic Carbon Efficiency Index. Abbreviations: NE, Northeast; N, North; NW, Northwest; E, East; CS, Central South; SW, Southwest.

The spatial distribution of the BCEI exhibits a clear decreasing gradient from southeast to northwest across China. The spatial distribution results are shown in Figure 3. High-value areas are mainly concentrated in the southeast and southwest where climatic conditions are favorable, with overall high BCEI values (above 0.00001 g/g C), indicating that while these ecosystems have a certain carbon sequestration capacity, they are also accompanied by a strong potential for ozone generation. Low-value areas, on the other hand, are distributed in the arid northwest and the high-altitude Tibetan Plateau. The zonal bar chart below the figure further quantifies this pattern, showing that the number of high BCEI values in East China (E) and Central–South (CS) far exceeds that in the Northwest (NW) and Northeast (NE). This finding strongly verifies the principle that China’s terrestrial ecosystem carbon storage is co-dominated by hydrothermal conditions and land cover types.

**Figure 4:**
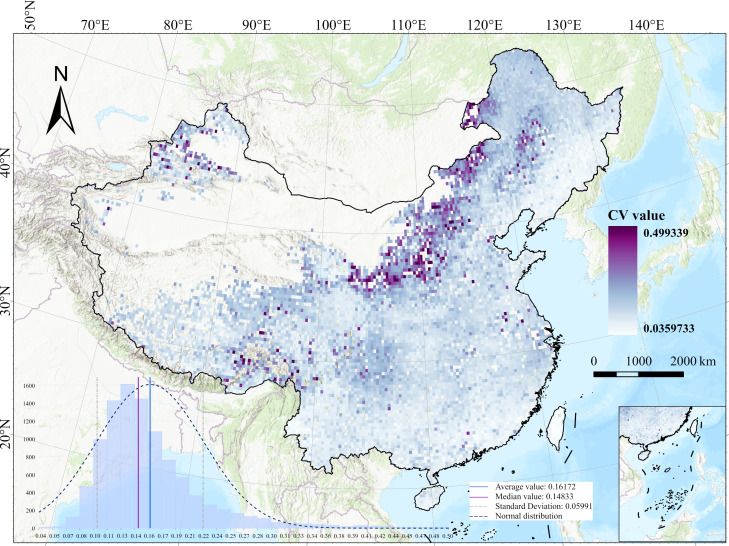
The pixel-wise Coefficient of Variation (CV) of BCEI values in China from 2001 to 2020. BCEI denotes the Biogenic Carbon Efficiency Index.

In this study, the pixel-wise Coefficient of Variation (CV) of BCEI values from 2001 to 2020 was calculated to reveal the spatial–temporal stability of vegetation change within the study area (Figure 4). As a metric for data dispersion, the CV effectively reflects the volatility of BCEI values over the time series. The results show that the CV of the BCEI exhibits significant spatial heterogeneity. Statistically, the median CV of the BCEI in the study area is 0.14 and the mean is 0.16, with a slightly right-skewed distribution. This indicates that BCEI values in most areas remained relatively stable over the 20-year period. However, a few pixels exhibit intense fluctuations, primarily in the northwestern part of the study area, as indicated by purple areas on the map, with CV values reaching about 0.4. These patchy or banded areas with high CV values indicate that the BCEI experienced drastic fluctuations during the study period. This statistical distribution is consistent with the temporal variation patterns commonly observed in vegetation indices in ecological research and falls within a reasonable range, which indirectly validates the reliability of the BCEI calculation results. The standard deviation of the CV ranges from 0.1 to 0.22, further quantifying the significant spatial disparity in the rate of BCEI change across the region.

### 2.3. Spatiotemporal Analysis and Future Trends

**Figure 5:**
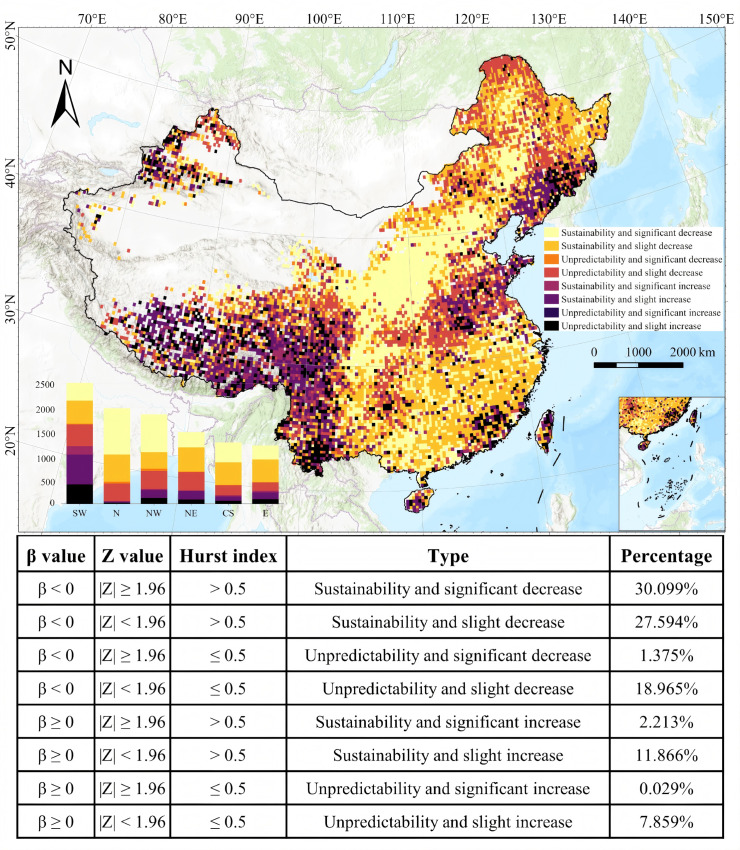
Spatiotemporal dynamics and sustainability classification of the BCEI Hurst index across China from 2001 to 2020. BCEI denotes the Biogenic Carbon Efficiency Index.

By combining the trend analysis with the Hurst exponent, this study reveals the spatial–temporal evolution and future trends of the BCEI from 2001 to 2020 (Figure 5). Overall, approximately 78% of the area showed a decreasing trend. Based on the Hurst exponent, 57.7% of the total area is projected to have a sustainable decrease, reflecting high ecosystem stability with gradual BCEI changes. A significant decreasing trend was observed in 31.5% of the area. Conversely, 22% of the area showed an increasing trend, although only 2.2% was significant. These areas of increase are mainly concentrated on the Tibetan Plateau (SW region), and the Shandong and Liaoning Peninsulas. This suggests that these regions may continue to release more BVOCs in the future and thus warrant further attention.

### 2.4. Driving Analysis

#### 2.4.1. Independent Effects of Factors

To clarify the driving factors of the BCEI and their regional differences, this study utilized the factor detector method from the geographical detector model to calculate and compare the q-statistic values for 10 factors (X1–X10) at both national and regional scales (Table 1). This approach quantifies the relative importance of each factor and reveals their explanatory power on the spatial differentiation of the BCEI. All factors were found to significantly influence the synergistic development of BVOCs and GPP at all regional scales (*p* < 0.01), with the results shown in the table. Due to the large scale of the study area and the complexity of the variables, a factor was considered dominant in explaining the spatial variation if its q-statistic was greater than 0.1.

At the national scale, temperature (X1, q = 0.1390), precipitation (X2, q = 0.1418), evapotranspiration (X4, q = 0.1323), and soil moisture (X5, q = 0.1184) were the dominant factors, indicating that the BCEI is primarily influenced by natural environmental factors across the country. At the regional scale, the dominance of factors showed significant differentiation. The single-factor geographical detector results revealed that in the E region, the q-values for all factors except evapotranspiration (X4) and grassland cover (X8) exceeded 0.1, with arid index (X6, q = 0.3038), precipitation (X2, q = 0.2977), and soil moisture (X5, q = 0.2968) being particularly prominent, reflecting the region’s high sensitivity to water-related factors. In the NE region, soil moisture (X5, q = 0.1593), forest cover (X7, q = 0.1304), and cropland cover (X10, q = 0.1366) were the dominant factors, indicating a combined influence of soil water and land use distribution on ecological processes. In the NW region, evapotranspiration (X4, q = 0.2360) and arid index (X6, q = 0.1558) were the main drivers, highlighting the key role of the arid climate in the region’s ecosystem. In the SW region, temperature (X1, q = 0.2034) and evapotranspiration (X4, q = 0.1448) were the dominant factors, suggesting that BCEI variations are co-regulated by thermal and water conditions. In contrast, the q-values in the N and CS regions were generally low with no obvious dominant factors, which may be due to their relatively balanced climate conditions or the presence of a single overriding factor, thus limiting the relative explanatory power of individual variables.

#### 2.4.2. Interactive Effects of Factors

**Figure 6:**
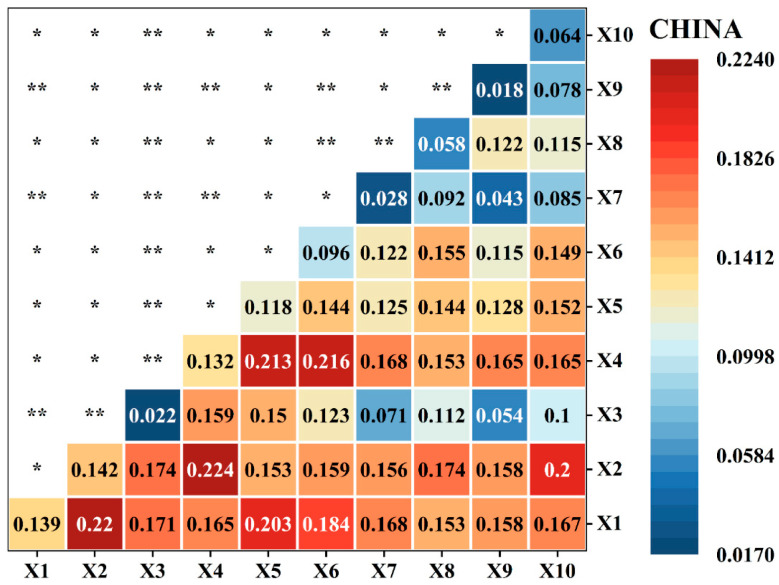
The q-values of factor interaction in China. Notes: * and ** indicate bivariable enhanced and nonlinear-enhanced, respectively. Note: X1: temperature; X2: precipitation; X3: Solar Radiation; X4: Evapotranspiration; X5: Soil Moisture; X6: Arid index; X7: Forest Cover Fraction; X8: Shrub Cover Fraction; X9: Grassland Cover Fraction; X10: Cropland Cover Fraction.

This study utilizes the interaction detector to calculate the interaction intensity among 10 variables, quantifying the combined effect of two or more variables on the target variable (Figure 6). The interaction results for all variable combinations were of an enhanced type, specifically bivariate enhancement (*) and nonlinear enhancement (**).

At the national scale, the results from the interaction detector show that the spatial heterogeneity of the BCEI is significantly influenced by the synergistic effects of multiple driving factors. Among these, 28 pairs exhibited bivariate enhancement and 17 pairs showed nonlinear enhancement, indicating a clear synergistic effect among these factors, where their combined explanatory power is greater than the sum of their independent effects. Notably, the interaction between evapotranspiration (X4) and precipitation (X2) was the strongest, reaching 0.224, followed by the interaction between temperature (X1) and precipitation (X2) at 0.22. Subsequently, the interactions of evapotranspiration (X4) with the soil moisture (X5) and arid index (X6) reached 0.21.

**Figure 7:**
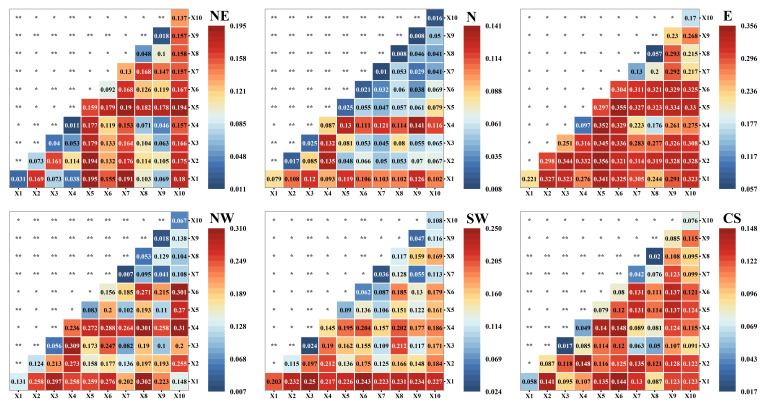
The q-values of factor interaction in different regions. Notes: * and ** indicate bivariable enhanced and nonlinear-enhanced, respectively. Note: X1: temperature; X2: precipitation; X3: solar radiation; X4: evapotranspiration; X5: soil moisture; X6: arid index; X7: forest cover fraction; X8: shrub cover fraction; X9: grassland cover fraction; X10: cropland cover fraction.

From Figure 7, in the E and NW regions, the bivariate interaction effects were most significant, with peak q-statistic values reaching approximately 0.3, reflecting strong composite mechanisms. Specifically, in the E region, the q-values were significantly enhanced both among climatic environmental factors and through their interactions with vegetation change factors. In the NW region, temperature (X1) and evapotranspiration (X4) exhibited multi-dimensional coupling enhancement effects with other natural climatic factors, all showing high interaction q-values. This indicates that the ecosystem in this region is highly sensitive to the combined changes in natural climatic factors. In the NE and SW regions, significant but moderately strong interaction effects were also detected, with maximum q-values reaching about 0.2. In the NE region, the interactions of soil moisture (X5), forest cover (X7), and cropland cover (X10) with other factors were significantly enhanced, suggesting that the key characteristics of ecological processes in this region are co-determined by aridity and vegetation type. In the SW region, the interaction effects were mainly manifested in the combination of temperature (X1) with other factors, which may be related to the region’s high environmental response sensitivity to temperature changes. Meanwhile, the SW region also revealed a linkage mechanism between shrubland (X8) and solar radiation (X3) and evapotranspiration (X4). This reflects that shrub vegetation is not merely a passive responder to climate but an active participant in regulating surface energy and water cycles, and its dynamics are key to understanding the ecosystem’s response to climate fluctuations in this area.

In contrast, the interaction effects in the CS and N regions were relatively weak, with maximum q-values of approximately 0.14. This indicates that the combined explanatory power of environmental factors for the target ecological variable is limited in these regions. Such weak interaction effects may be associated with relatively homogeneous climatic conditions or the dominance of a single controlling factor, which reduces the potential for synergistic enhancement among multiple drivers.

## 3. Discussion

### 3.1. Interregional Differences in the Spatiotemporal Patterns of BCEI

Our results show that, in terms of temporal trends, the BCEI exhibits relatively modest overall variability. By contrast, pronounced spatial heterogeneity is evident, with a broad gradient of decreasing values from the humid southeastern coast toward the arid northwestern interior. This pattern closely aligns with regional differences in water availability and vegetation types. We therefore use 2020 as a representative year to analyze spatial heterogeneity and probe the underlying ecological mechanisms. By comparing the BCEI across regions within their dominant land cover types (Table 2), we further elucidate structural, ecological controls that shape regional contrasts, thereby deepening understanding of the coupling between carbon sequestration and ozone-forming potential.

First, the BCEI levels in the E and CS regions are the most prominent in the country, primarily due to the high performance of the forest and shrubland ecosystems within these regions. These regions are dominated by subtropical evergreen broad-leaved forests, which feature complex community structures, high biodiversity, and superior hydrothermal conditions, leading to nationally leading GPP levels. However, many dominant tree species (such as those from the Lauraceae and Magnoliaceae families) are inherently high BVOC emitters [32]. It should be noted that the BVOC estimates used in this study are derived from plant functional type (PFT)-based parameterizations rather than species- or family-level emission factors; the reference to these families is therefore intended as a physiological interpretation consistent with the dominant broad-leaved evergreen PFTs represented in the model. Additionally, the hot and humid environment promotes continuous physiological activity throughout the year, which disproportionately amplifies ozone formation potential (OFP) output, with its growth elasticity exceeding that of GPP. Therefore, despite their strong carbon sink capacity [33], the environmental regulation function (or pollution potential) corresponding to each unit of carbon sink is higher, leading to BCEI values that are higher than in other regions.

In contrast, the overall BCEI levels in the Northeast (NE) and Southwest (SW) regions are relatively low, which is primarily influenced by the grassland ecosystems within these areas. The BCEI value for grasslands in the NE region (0.800) is the lowest in the country. This may be because the species composition of temperate grasslands is relatively simple, leading to an inherently weak capacity for OFP (e.g., BVOC emissions). Although its GPP is limited by a short growing season [34], its OFP level is even lower [35], resulting in a smaller BCEI ratio. Similarly, grasslands in the SW region (1.072) also exhibit a low BCEI. The subtropical grasslands in this region have high productivity (GPP) under humid and hot conditions, but the BVOC emission intensity of herbaceous plants is far less than that of woody plants, forming a “high GPP, low OFP” combination that lowers the BCEI value.

Notably, the Northwest (NW) region shows significant functional differentiation internally, with BCEI values for cropland (1.937) and shrubland (1.741) being among the highest in the country, while that for forest (1.354) is relatively low. This phenomenon reflects the different adaptation strategies of ecosystems under water stress. In arid environments, shrubland, as native vegetation, has evolved mechanisms to cope with stress by releasing BVOCs, thus maintaining a high OFP level [36,37], while its GPP is limited by water scarcity, leading to a high BCEI. Cropland dependent on irrigation exhibits a similar pattern of low GPP and relatively high OFP. However, the forests in this region are mostly drought-resistant coniferous forests, where severe water stress comprehensively suppresses physiological activities such as photosynthesis and BVOC synthesis [38], keeping the overall BCEI level within a lower range.

These results further suggest that region-differentiated vegetation planning could enhance the practical value of the BCEI. In high-BCEI regions (e.g., East China and Central–South China), where forest and shrubland ecosystems dominate BCEI levels, incorporating BVOC emission traits into afforestation and greening decisions may reduce ozone formation potential per unit carbon fixed. In practice, this can be operationalized by prioritizing locally adapted, lower-BVOC-emitting species or mixed stands with a reduced dominance of high-emitting functional groups in ozone-sensitive areas, while limiting the expansion of well-documented high-isoprene emitters (e.g., poplars, willows, and oaks) in densely populated corridors. Meanwhile, such vegetation-planning measures may also contribute to the observed long-term BCEI decline. Beyond hydroclimatic controls, the nationwide decrease in the BCEI over 2001–2020 likely reflects the combined influence of climatic change and land management policies. From a climatic perspective, CO_2_ fertilization and warming-induced growing season extension can enhance photosynthetic carbon uptake, potentially increasing GPP more rapidly than BVOC-related ozone formation, thereby lowering the BCEI. In parallel, large-scale afforestation and reforestation programs have altered vegetation structure and species composition in many regions, in some cases favoring fast-growing or relatively low-emitting species, which may further suppress ozone formation potential per unit carbon fixed. Although disentangling the relative contributions of climate-driven and policy-driven effects remains challenging at the national scale, their concurrent influence provides a plausible explanation for the sustained BCEI decline observed in this study. Such species-screening measures, implemented through region-specific emission factor databases and local observations rather than universal prescriptions, would help improve the carbon–air quality co-benefits of greening initiatives.

### 3.2. Associations of Hydroclimatic Variables with BCEI

Results from the Geographical Detector indicate that temperature, precipitation, evapotranspiration, and soil moisture have the strongest explanatory power for the BCEI at the national scale. As these hydroclimatic variables dominate BCEI’s spatial variability, we analyze their correlations with the BCEI and map the associated spatial patterns.

**Figure 8:**
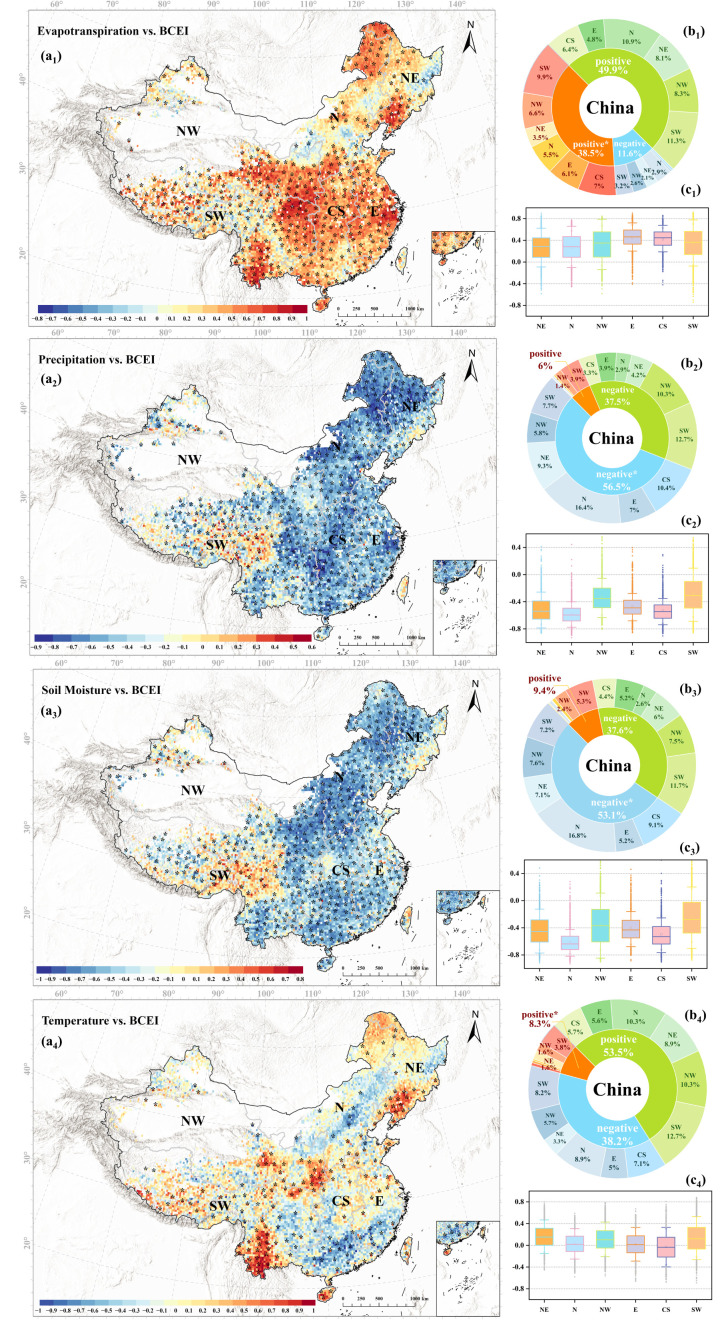
Spatial distribution of the correlations between BCEI and important influencing factors from 2001 to 2020. Note: (**a_1_**–**a_4_**) Show the spatial distribution of correlation coefficients (RBCEI-Evapotranspiration, RBCEI-Precipitation, RBCEI-Soil Moisture and RBCEI-Temperature) between BVOC carbon efficiency index (BCEI) and evapotranspiration, precipitation, soil moisture and temperature for the entire study period. * indicate significant Spearman correlations with *p*  <  0.05; (**b_1_**–**b_4_**), (**c_1_**–**c_4_**) are the statistical distributions of RBCEI-Evapotranspiration, RBCEI-Precipitation, RBCEI-Soil Moisture and RBCEI-Temperature for NE, N, E, NW, SW and CS regions, respectively. The maximum and minimum extents of the colored boxes indicate the 25th and 75th percentiles and the whiskers show the outliers, which are below the 5th percentile and above the 95th percentile.

The results show that all three water-related factors are significantly correlated with the BCEI, but their directional effects and spatial response characteristics differ markedly. First, evapotranspiration (ET) shows a generally significant positive correlation with the BCEI, with 38.5% of pixels nationwide exhibiting a significant positive correlation, and the median correlation coefficient in each region is approximately 0.4 (Figure 8(a_1_)). This result indicates that ET has a promoting effect on the BCEI. From a physiological perspective, evapotranspiration stress directly regulates stomatal conductance, and when evapotranspiration stress intensifies, plants tend to partially close their stomata. This action limits CO_2_ entry, causing GPP growth to slow or even decline. However, even under these conditions, plants continue to transpire, maintaining crucial water and nutrient cycles (sustaining OFP). Although stomatal closure and metabolic limitations inhibit the release of BVOCs [39,40], it also exacerbates the feedback between soil water deficit and atmospheric dryness, restricting vegetation transpiration and the fraction of photosynthetically active radiation used for light use efficiency, thereby weakening GPP accumulation [41]. Furthermore, drought may disrupt the coupling relationship between LAI and GPP, thus indirectly affecting the trend of the BCEI [42,43]. This asymmetric stomatal control over carbon assimilation and BVOC-related ozone formation implies that as ET increases, the growth of OFP may be more significant than that of GPP, ultimately increasing the BCEI.

In contrast, an improvement in water supply (such as increased soil moisture and precipitation) typically helps to synchronously enhance both BVOC emissions and GPP levels. The analysis shows that both soil moisture and precipitation are significantly negatively correlated with the BCEI, with 53.1% and 56.5% of the regions showing significant negative correlations, respectively, and a median correlation coefficient of approximately −0.4 for both (Figure 8(b_2_,_b3_,_c2_,_c3_)). This reveals a key ecological trade-off: in water-driven ecosystems, the growth elasticity of GPP is much greater than that of OFP. Mechanistically, in regions where water is the primary limiting factor, once water conditions improve, the ecosystem rapidly allocates resources preferentially to photosynthesis, leading to an explosive growth in GPP. This response is largely mediated by stomatal reopening under improved water availability, which disproportionately enhances CO_2_ uptake relative to BVOC emission increases. Studies have pointed out that GPP is extremely sensitive to changes in soil moisture, especially in arid and semi-arid regions, where water limitation can cause a global GPP reduction of up to 40% by suppressing fLUE [44], and precipitation is also one of the dominant factors in GPP inter-annual variability [45]. In comparison, although improved water conditions also promote BVOC emissions [20], their growth magnitude may not match that of GPP. On one hand, the emission of some BVOCs (like monoterpenes) is related to the plant’s stress state [46]; when water stress is relieved, the emission of such BVOCs may not increase or may even decrease. On the other hand, other complex ecosystem functions, such as the perfection of nutrient cycles and the recovery of biodiversity, typically respond more slowly than basic productivity.

Temperature exhibits different correlations in different regions (Figure 8(a_4_)). For instance, significant positive correlation clusters are found in areas like Yunnan, Liaoning, Shaanxi, and Heilongjiang. This spatial heterogeneity depends on whether the temperature of an ecosystem is below or above its optimal range for physiological activity, and how temperature changes interact with other conditions (especially water). In regions with favorable hydrothermal conditions, such as Southeast China, the temperature has already reached or exceeded the optimal range. In this case, warming acts as a high-temperature stress, leading to a negative correlation with the BCEI, primarily by increasing respiratory consumption and inhibiting photosynthetic activity. Furthermore, in water-limited areas like North and Northwest China, warming exacerbates water stress by increasing evaporative demand, forcing plants to close their stomata, which leads to a comprehensive decline in both GPP and OFP functions, similarly causing a decrease in the BCEI. In regions like Yunnan and Liaoning, where the temperature is below the optimal range, warming is a benefit, primarily activating vegetation by relieving low-temperature limitations and extending the growing season. Moreover, OFP and GPP exhibit an asymmetric response to warming; once temperature is no longer the primary limiting factor, GPP growth is more quickly constrained by other factors like water and light, while the enhancement of OFP is more sustained, thereby increasing the BCEI.

### 3.3. Limitations and Future Works

Although this study systematically evaluated the coupling between BVOC emissions and GPP across regions of China from 2001 to 2020 and revealed the spatiotemporal characteristics of the BCEI from the dual perspectives of carbon sequestration and air quality, several aspects warrant further refinement and extension.

First, the spatial resolution mismatch among datasets may affect analytical precision. The BVOC dataset used in this study has a resolution of approximately 27 km, which is notably coarser than the 1 km or finer resolution of GPP and climate variables. This disparity limits spatial detail, particularly in topographically complex and ecologically heterogeneous regions, and may obscure localized patterns. Future research should consider incorporating higher-resolution BVOC simulations or remote sensing products to improve spatial accuracy.

Second, there is inherent uncertainty in the estimation of OFP. This study applied the maximum incremental reactivity (MIR) coefficients. MIR quantifies the incremental ozone formed per unit increase in a given VOC under standardized conditions. However, these coefficients were derived under specific experimental conditions based largely on atmospheric compositions in the United States. Their applicability to China’s diverse climatic and pollution contexts remains uncertain. Moreover, BVOC emission coefficients can vary substantially with species traits, phenological stage, and plant stress conditions (e.g., drought and heat extremes), which may further propagate uncertainty into BCEI estimates. Future studies should integrate China-specific observational data or modeling experiments to calibrate the BVOC–ozone reaction mechanisms, enhancing the scientific robustness and contextual relevance of OFP assessments.

Third, seasonal variation was not fully captured in this study. The analysis was conducted at an annual timescale, focusing on long-term trends. However, previous research has shown that BVOC emissions exhibit strong seasonality, with substantial increases during hot summer months. Future investigations should employ finer temporal resolutions (e.g., seasonal or monthly), incorporate meteorological variability, and explore multi-timescale BVOC-GPP dynamics to more accurately uncover underlying mechanisms and response pathways.

Finally, while the BCEI, defined as the ratio of BVOC emissions to GPP, effectively captures the relative intensity of carbon release versus carbon uptake, it does not reflect their absolute magnitudes. For instance, a BCEI value of 2 may arise from BVOC = 100 and GPP = 50, or BVOC = 80 and GPP = 40. Although the ratios are identical, the former scenario involves greater carbon flux and metabolic activity. Relying solely on a ratio may therefore mask regional differences in actual carbon exchange intensity. Moreover, the BCEI focuses exclusively on the BVOC–carbon relationship and does not account for other key dimensions of ecosystem services, such as vegetation restoration, hydrological impacts, or the integration of multiple objectives relevant to large-scale ecological programs like afforestation. To enhance its practical utility in ecological management and policymaking, future studies should explore ways to integrate the BCEI into broader ecosystem service frameworks.

## 4. Materials and Methods

### 4.1. Study Area

Located in eastern Eurasia, China covers approximately 9.6 million km^2^ and spans multiple climate zones and ecoregions. The landscape follows a three-step staircase from west to east and includes monsoonal humid, continental arid, and plateau–mountain climate regimes. Correspondingly, vegetation is highly diverse, encompassing eastern forests and croplands, northwestern grasslands and desert shrublands, and alpine meadows and shrublands on the Tibetan Plateau. As a global frontrunner in greening, China’s extensive vegetation restoration efforts are broadly representative. This strong spatial heterogeneity underpins varied carbon-cycling mechanisms and provides a unique natural setting for examining how BVOC emissions co-vary with GPP across space and time.

### 4.2. Data Sources and Processing

#### 4.2.1. BVOC Dataset

This study draws BVOC emissions from ECCAD—the Emissions of atmospheric Compounds and Compilation of Ancillary Data database. For the China domain, we analyzed annual BVOC fields with 0.25° spatial resolution spanning 2001–2020, which served to quantify their influence on ozone formation potential (OFP). The dataset provides emission fluxes in kg m^−2^ s^−1^.

#### 4.2.2. GPP Dataset

We used the MODIS Terra MOD17A2H (Version 6.1) GPP product. Based on a radiation use efficiency (RUE) model, MOD17A2H combines vegetation index observations and meteorological inputs to produce 8-day GPP composites at 500 m. To align with the BVOC emissions data, the GPP grids were resampled to 0.25° and composited to the annual scale (g C m^−2^ yr^−1^) for subsequent spatiotemporal assessments of ecosystem processes.

#### 4.2.3. Driving Factors

We selected ten representative and readily quantifiable drivers as independent variables, grouped into climatic environmental factors and vegetation change factors. The climatic environmental set comprises air temperature, precipitation, surface downward shortwave radiation, evapotranspiration, aridity, and soil moisture; the vegetation change set includes fractional cover of forests, shrubs, grasslands, and croplands. Air temperature, precipitation, evapotranspiration, and aridity were sourced from the National Tibetan Plateau/Third Pole Data Center (1 km; 1901–2023), whereas soil moisture was obtained from the FEWS NET Land Data Assimilation System (FLDAS; 0.1°; 1982–2023). Fractional covers of forest, shrubland, grassland, and cropland were derived from the land use/land cover (LULC) products of the National Ecosystem Science Data Center (10 km; 1980–2021). For this study, all datasets were subset to 2001–2020, reprojected to an Albers equal-area conic projection, and resampled in ArcGIS Pro 3.0 (Esri, Redlands, CA, USA) to 0.25° to match the analysis scale of the BVOC and GPP datasets. Details for each driver are summarized in Table S1.

### 4.3. Methods

Based on BVOC and GPP fields, we derive a BCEI to represent vegetation–BVOC coupling. BCEI trends for 2001–2020 are examined with the Sen-MK method, while the Hurst exponent is used to evaluate the persistence and foreseeability of future variations. We then employ the Geographical Detector to diagnose dominant controls on the BCEI’s spatial heterogeneity, and use Spearman rank correlations to assess relationships between major ecological covariates and the BCEI.

#### 4.3.1. Quantifying the Relationship Between BVOCs and GPP

• Calculation of OFP

BVOCs are key precursors of tropospheric ozone: in the presence of nitrogen oxides (NO_3_) and sunlight, they undergo photochemical reactions that form ozone [47]. Different VOC classes exhibit distinct ozone formation potentials (OFP), governed by both their reaction rate constants with oxidants and pathway mechanisms [48]. OFP is commonly defined as the mass of ozone produced per gram of VOC (g O_3_ g^−1^ VOC) and is widely used to assess the relative contributions of VOCs to tropospheric ozone formation. Among available metrics, the maximum incremental reactivity (MIR) is frequently employed to quantify ozone-forming propensity under specified environmental conditions and to derive compound-specific OFP values [49]. Using China-wide annual BVOC emissions for 2001–2020 together with the corresponding MIR coefficients, we compute BVOC-related OFP according to Equation (1).

(1) $$OFP=\sum {(C}_{{VOC}_{i}}\times \;{MIR}_{i})$$

Here, $C_{{VOC}_{i}}$ represents the measured concentration of ${VOC}_{i}$, and ${MIR}_{i}$ denotes the maximum incremental reactivity of ${VOC}_{i}$ (Table S2 in the Supplementary Materials), that is, the amount of O_3_ formed per unit increase of ${VOC}_{i}$ added to a representative atmospheric mixture.

• Calculating the Index of Coordinated Development Level

We developed the BCEI as an integrated metric to characterize the trade-off between ecosystem carbon sequestration and ozone pollution potential. Conceptually, the BCEI brings the ecosystem’s positive contribution (carbon fixation) and its potential adverse effect (ozone formation) into a unified assessment framework, thereby providing a new composite criterion for ecological security appraisal and coordinated environmental governance. The index is defined as follows in Equation (2):

(2) $$BCEI=\frac{OFP}{GPP}$$

The unit of the BCEI is g/g C, representing the OFP associated with each unit of carbon fixed by vegetation. It reflects the balance between carbon uptake and atmospheric pollution within ecosystems. Physically, it can be interpreted as the potential amount of O_3_ generated per gram of carbon sequestered. Lower BCEI values indicate lower ozone formation potential per unit carbon fixed and therefore correspond to greater ecological efficiency, reflecting a desirable “high carbon sequestration and low pollution” synergy. Conversely, a higher BCEI value suggests a greater ozone formation potential per unit of carbon fixed. Although such systems may exhibit strong carbon sink capacity, they also pose higher environmental pollution risks, limiting their overall ecological benefits. Therefore, the BCEI can help identify regions where elevated carbon costs are accompanied by significant pollution risks, as well as ecosystems that demonstrate more favorable carbon–pollution synergy. This index provides a quantitative basis and scientific support for ecological engineering planning, vegetation type selection, and regional air quality management.

#### 4.3.2. Trend Analysis

The Theil–Sen median trend estimator and the Mann–Kendall (MK) test were applied to assess long-term BCEI trajectories. The Theil–Sen method, also known as Sen’s slope estimator, is a robust non-parametric approach for quantifying monotonic trends [50]. This method is computationally efficient and insensitive to measurement errors and outliers, making it well suited for trend detection in long-term time series.

(3) $$\beta =Median\left(\frac{R_{j}-R_{i}}{j-i}\right),2001\leq i\;<\;j\;\leq \;2020$$

Here, i and j denote the time sequence indices, and $R_{i}$ and $R_{j}$ represent the grid unit ratio values at times i and j, respectively. A value of β>0 indicates an upward trend over time, β<0 indicates a downward trend, and β=0 suggests a stable or no-trend condition.

Given that β is a non-standardized parameter, it only reflects the magnitude of trend change within the time series itself. However, the significance of the trend cannot be evaluated based on β alone. Therefore, the Mann–Kendall (MK) test is employed to statistically assess whether the observed trend in the ratio values is meaningful. The Mann–Kendall test is a non-parametric statistical method originally proposed by Mann in 1945 and later refined by Kendall and Sneyers [51]. Its advantages include not requiring the data to follow a normal distribution, not assuming a linear trend, and being robust to missing values and outliers. As a result, it has been widely applied in the significance testing of long-term trends in environmental and climatological time series. The Mann–Kendall test statistic *S* is calculated using Equations (4)–(7):

(4) $$S=\sum_{i=1}^{n-1}\sum_{j=i+1}^{n}sgn\left(R_{j}-R_{i}\right)$$

(5) $$sgnR_{j}-R_{i}=\left\{\begin{array}{c} \;\;\;\;\;1,\;\;\;\;\;\;R_{j}-R_{i}>0\\ \;\;\;\;\;0,\;\;\;\;\;\;R_{j}-R_{i}=0\\ \;-1,\;\;\;\;\;R_{j}-R_{i}<0 \end{array}\right.$$

(6) $$Var\left(S\right)=\frac{n\left(n+1\right)\left(2n+5\right)}{18}$$

(7) $$Z=\left\{\begin{array}{c} \frac{S-1}{\sqrt{Var\left(S\right)}},\;\;\;\;\;\;S>0\\ 0\;\;\;\;\;\;\;\;\;\;\;\;\;\;,\;\;\;\;\;\;\;S=0\\ \frac{S+1}{\sqrt{Var\left(S\right)}},\;\;\;\;\;\;S<0 \end{array}\right.$$

Here, n denotes the number of observations in the time series; $R_{i}$ and $R_{j}$ represent the test values at time steps i and j (where j > i) and $\mathrm{sgn}(R_{j}-R_{i})$ is the sign function used in the Mann–Kendall test.

Z is the standardized test statistic, with values ranging from −∞ to +∞. A trend is considered statistically significant at a given significance level α if $|Z|>u_{1-\alpha /2}$ where $u_{1-\alpha /2}$ is the critical value from the standard normal distribution. In this study, the significance level was set at α = 0.05. Accordingly, a $\left|Z\right|$ value greater than 1.96 indicates that the trend passes the significance test at the 95% confidence level.

#### 4.3.3. Hurst Exponent

The Hurst exponent characterizes self-similarity and long-term dependence in natural processes [52]. It has been widely adopted in time series analysis to measure long-term memory and has proven effective in studies of long-term vegetation dynamics. The value of the Hurst exponent ranges from 0 to 1: If 0 < H < 0.5, the time series is anti-persistent, suggesting that future values are likely to move in the opposite direction of past trends. If H = 0.5, the time series behaves like a random walk, indicating no correlation between past and future values and thus no predictability. If 0.5 < H < 1, the series is persistent, implying that future changes are likely to follow the same trend direction. In general, a lower H value indicates greater unpredictability, whereas a higher H value suggests stronger trend persistence and long-term sustainability.

#### 4.3.4. Coefficient Variability Stability Analysis

The Coefficient of Variation (CV) reflects the relative degree of fluctuation in the BCEI. A larger value indicates a greater intensity of disturbance and higher instability of the BCEI [53]. The CV is calculated as follows:

(8) $$CV=\frac{\sqrt{\frac{1}{n-1}\sum_{i=1}^{n}{\left({BCEI}_{i}-{BCEI}_{mean}\right)}^{2}}}{{BCEI}_{mean}}$$

CV represents the Coefficient of Variation in the BCEI, n is the length of the time series, BCEIi represents the BCEI value for year i, and BCEImean is the multi-year average BCEI value from 2001 to 2020.

#### 4.3.5. Geographical Detector

Geodetector is a statistical method used to identify spatial heterogeneity and to uncover the underlying factors driving geographic variation.

• Factor detector

(9) $$q=1-\frac{\sum_{h=1}^{L}N_{h}\delta_{h}^{2}}{N\delta^{2}}$$

The q-statistic in Geodetector measures the explanatory power of a driving factor X on the spatial distribution of a dependent variable. The value of q ranges from 0 to 1, with higher values indicating a stronger explanatory power of factor X over the dependent variable. Both the dependent and independent variables can be stratified into h discrete categories, where h = 1, 2, …, L, …, Nh denotes the number of units in stratum h, N is the total number of units in the study area, $\delta_{h}^{2}$ represents the variance of the dependent variable within stratum h, and $\delta^{2}$ is the variance of the dependent variable over the entire study area.

• Interaction detector

The interaction detector in Geodetector is used to assess the combined explanatory power of two factors, $X_{i}$ and $X_{j}$, on a dependent variable (Table S3 in the Supplementary Materials). The procedure involves three steps: First, compute the individual q-values of the two factors: q($X_{i}$) and q($X_{j}$). Second, calculate the q-value of their interaction: q($X_{i}\cap X_{j}$). Last, compare q($X_{i}$), q($X_{j}$) and q($X_{i}\cap X_{j}$) to determine the nature and strength of the interaction effect [54].

## 5. Conclusions

Using BVOC emissions (2001–2020) and MODIS GPP, we constructed the biogenic carbon efficiency index (BCEI) and employed the Sen–MK test and Hurst exponent to characterize its temporal evolution. We then mapped nationwide spatiotemporal patterns and applied Geodetector to identify dominant drivers, complemented by Spearman correlations to examine hydrothermal controls. The main findings are: First, the BCEI shows a weak nationwide decline over 2001–2020. Spatially, the BCEI exhibits pronounced regional differentiation, with the highest annual means in the East (E) and Central–South (CS) regions, forming a broad gradient decreasing from the humid, climatically favorable Southeast toward the Northwest. High-value clusters are concentrated in the Southeast and Southwest. Second, approximately 78.032% of China shows a declining BCEI trend, and 57.693% is characterized by sustained decline, indicating improved carbon use efficiency and strengthened sequestration potential. In contrast, increases are mainly observed over the Southwest Tibetan Plateau and the Shandong and Liaodong Peninsulas. Finally, temperature, evapotranspiration (ET), soil moisture and precipitation emerge as the core nationwide drivers, underscoring the central role of hydrothermal conditions in governing ecosystem allocation efficiency. Their correlations with the BCEI differ: at the national scale, soil moisture and precipitation are predominantly negatively correlated with the BCEI, ET is positively correlated, and temperature exhibits region-dependent relationships.

These results clarify how hydroclimatic regimes mediate the trade-off between carbon sequestration efficiency and BVOC emissions across ecological zones, providing an evidence base for region-specific ecosystem management. Nevertheless, applicability is conditioned by the BCEI’s ratio nature, cross-dataset resolution gaps, and the need for localized parameters. Future work will refine the BCEI construct, increase data resolution, and extend the framework globally to support multi-objective vegetation management.

## Figures and Tables

**Table 1:** The q-value of the driving factors across different regions in China.

	CHINA	NE	N	E	NW	SW	CS
X1	0.1390	0.0308	0.0787	0.2207	0.1310	0.2034	0.0576
X2	0.1418	0.0732	0.0169	0.2977	0.1244	0.1149	0.0872
X3	0.0217	0.0397	0.0250	0.2506	0.0564	0.0239	0.0170
X4	0.1323	0.0110	0.0872	0.0969	0.2360	0.1448	0.0486
X5	0.1184	0.1593	0.0251	0.2968	0.0830	0.0900	0.0790
X6	0.0964	0.0925	0.0211	0.3038	0.1558	0.0618	0.0797
X7	0.0281	0.1304	0.0097	0.1300	0.0065	0.0359	0.0424
X8	0.0580	0.0482	0.0083	0.0567	0.0535	0.1171	0.0204
X9	0.0185	0.0177	0.0078	0.2298	0.0179	0.0470	0.0855
X10	0.0641	0.1366	0.0160	0.1705	0.0675	0.1079	0.0759

X1: temperature; X2: precipitation; X3: solar radiation; X4: evapotranspiration; X5: soil moisture; X6: arid index; X7: forest cover fraction; X8: grassland cover fraction; X9: shrub cover fraction; X10: cropland cover fraction. The q-value indicates how much of the spatial variation in the dependent variable is explained by a given driving factor, with higher values representing stronger explanatory power.

**Table 2:** The average values of BCEI (10^−5^ g/g C) for different vegetation types. BCEI denotes the Biogenic Carbon Efficiency Index.

	Crop	Grass	Forest	Shrub	Total
NE	1.044	0.800	1.782	1.483	5.110
N	1.558	1.401	1.585	1.340	5.884
E	1.725	-	3.227	3.527	8.479
NW	1.937	1.258	1.354	1.741	6.291
SW	1.443	1.072	1.486	1.517	5.517
CS	2.120	-	2.504	2.539	7.162

## Data Availability

Data will be made available on request.

## Supplementary Materials

The following supporting information can be downloaded at: https://www.mdpi.com/article/10.3390/plants15040564/s1, Table S1. Influencing factors of BVOC Emissions and GPP Coupling. Table S2. Maximum Incremental Reactivity (MIR, g O_3_/g VOC) values for selected BVOC species and their representative substitutes. Table S3. Types of interaction between two covariates.
