# Supplementary material for: Spatial–Temporal Heterogeneity and Driving Mechanisms of the Relationship Between Vegetation Carbon Sequestration and Biogenic Volatile Organic Compounds (BVOC) Emissions in China
Source: Plants (Basel). 2026 Feb 11;15(4):564. doi: 10.3390/plants15040564 (PMC12943996; doi:10.3390/plants15040564)
Supplement: Supplementary file 1 [file plants-15-00564-s001.zip › plants-4078186-supplementary.pdf]

Supplementary Material

Spatial-temporal heterogeneity and driving mechanisms of the relationship between vegetation carbon sequestration and BVOC emissions in China

Table S1

Influencing factors of BVOC Emissions and GPP Coupling.

	Driving factors	Code	Unit	Website
Climatic & Environmental Conditions	Temperature	X1	℃	https://data.tpdc.ac.cn
	Precipitation	X2	mm	
	Solar Radiation	X3	0.5°	https://www.climatologylab.org
	Evapotranspiration	X4	mm	https://data.tpdc.ac.cn
	Soil Moisture	X5	m^3/m^3	https://ldas.gsfc.nasa.gov
	Arid Index	X6	-	https://data.tpdc.ac.cn
Vegetation change	Forest Cover Fraction	X7	%	https://www.nesdc.org.cn
	Grassland Cover Fraction	X8		
	Shrub Cover Fraction	X9		
	Cropland Cover Fraction	X10		

Note: To simplify subsequent descriptions, each driving factor is assigned a code due to the large number of variables involved.

Table S2

Maximum Incremental Reactivity (MIR, g O_3/g VOC) values for selected BVOC species and their representative substitutes.

BVOC species	MIR (g O_3/g VOC)	BVOC species	MIR (g O_3/g VOC)
acetone	0.35	methanol	0.65
acetaldehyde	6.34	methyl-bromide	0.018
butanes-and-higher-alkanes	1.08	methyl-chloride	0.036
butenes-and-higher-alkenes	9.42	methyl-iodide	-0.53
CH_4	0.014	other-aldehydes	4.18
CO	0.053	other-ketones	4.48
ethane	0.26	other-monoterpenes	6. 16
ethanol	1.45	pinene-a	4.38
ethene	8.76	pinene-b	3.38
formaldehyde	9.24	propane	0.46
hydrogen-cyanide	0.2	propene	11.37
isoprene	10.28	sesquiterpenes	4.01
MBO	4.73	toluene	3.88

Note: For certain BVOC categories, representative compounds were used to approximate MIR values due to the lack of specific data. The substitutions are as follows: Butanes and higher alkanes are represented by n-butane; Butenes and higher alkenes are represented by n-butene; Other aldehydes are represented by hexanal; Other ketones are represented by isophorone; Other monoterpenes are represented by terpinolene; Sesquiterpenes are represented by sabinene.

Table S3

Types of interaction between two covariates

Interaction relationship	Interaction type
$q(X_i \cap X_j) < Min\left(q(X_i), q(X_j)\right)$	Nonlinear-weaken
$Min\left(q(X_i), q(X_j)\right) < q(X_i \cap X_j) < Max\left(q(X_i), q(X_j)\right)$	Uni-variable weaken
$q(X_i \cap X_j) > Max\left(q(X_i), q(X_j)\right)$	Bivariable enhanced
$q(X_i \cap X_j) = q(X_i) + q(X_j)$	Independent
$q(X_i \cap X_j) > q(X_i) + q(X_j)$	Nonlinear-enhanced
